# Feasibility of automated target centralization in colonoscopy

**DOI:** 10.1007/s11548-015-1301-3

**Published:** 2015-10-08

**Authors:** N. van der Stap, E. D. Rozeboom, H. J. M. Pullens, F. van der Heijden, I. A. M. J. Broeders

**Affiliations:** Department of Robotics and Mechatronics, MIRA Institute, University of Twente, Carré 3.625, P. O. Box 1502, 7500 AE Enschede, The Netherlands; Department of Gastroenterology and Hepatology, Meander Medical Center, Amersfoort, The Netherlands; Department of Surgery, Meander Medical Center, Amersfoort, The Netherlands

**Keywords:** Robotized endoscopy, Image-based endoscope navigation, Automated endoscopy, Colonoscopic interventions

## Abstract

**Purpose:**

Early detection of colorectal cancer is key to full recovery. This urged governments to start population screening programs for colorectal cancer, often using flexible endoscopes. Flexible endoscopy is difficult to learn and time-consuming. Automation of flexible endoscopes may increase the capacity for the screening programs. The goal of this pilot study is to investigate the clinical and technical feasibility of an assisting automated navigation algorithm for a colonoscopy procedure.

**Methods:**

Automated navigation (lumen centralization) was implemented in a robotized system designed for conventional flexible endoscopes. Ten novice and eight expert users were asked to perform a diagnostic colonoscopy on a colon model twice: once using the conventional and once using the robotic system. Feasibility was evaluated using time and location data as measures of the system’s added value.

**Results:**

Automated target centralization (ATC) was turned on by the novices for a median of 4.2 % of the time during insertion and 0.3 % during retraction. Experts turned ATC on for 4.0 % of the time during insertion and 11.6 % during retraction. Novices and experts showed comparable times to reach the cecum with the conventional or the robotic setup with ATC.

**Conclusion:**

The ATC algorithm combined with the robotized endoscope setup works in an experimental setup that closely resembles the clinical environment and is considered feasible, although ATC use was lower than expected. For novices, it was unclear whether the low usage was due to unfamiliarity with the system or because they did not need ATC. Experts used ATC also during the retraction phase of the procedure. This was an unexpected finding and may indicate an added value of the system.

**Electronic supplementary material:**

The online version of this article (doi:10.1007/s11548-015-1301-3) contains supplementary material, which is available to authorized users.

## Introduction

**Fig. 1:**
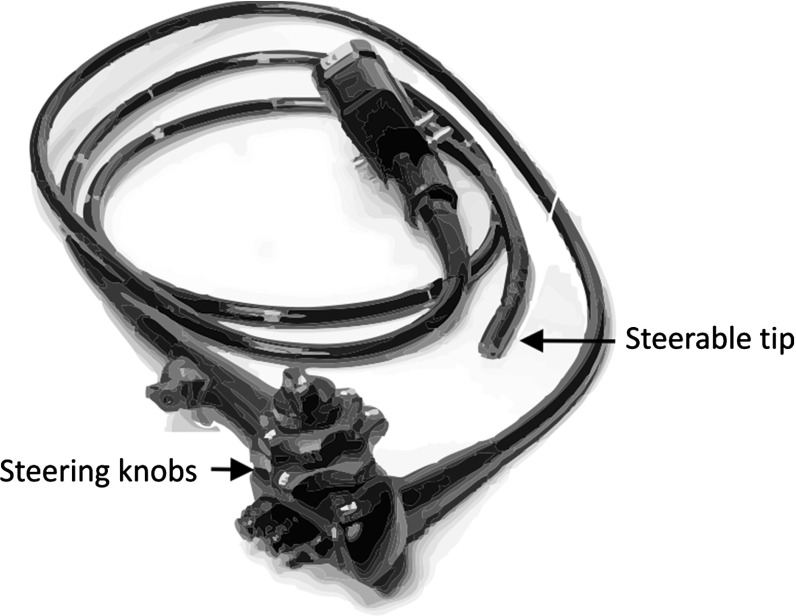
A typical colonoscope with two steering knobs which are used to steer the tip up/down or left/right

Colorectal cancer has one of the highest incidences of all cancers in the Western world [1]. Colonoscopy, inspection of the colon with an endoscope (Fig. 1), is a vital tool in the screening procedure for colorectal cancer. It is a useful next step after a positive fecal occult blood test (FOBT), which is the first step in national screening programs in many European countries [2]. In the Netherlands, a national population screening program was started in 2014. This program is expected to increase the number of colonoscopies by 70,000 yearly [3]. Currently, approximately 190,000 colonoscopies are performed each year, which implies an increase in demand of over 35 % [4, 5]. Controlling the endoscope is difficult to learn; starting endoscopists require a learning curve of 100–300 procedures to reach competency in colonoscopy [6]. Colonoscopes are steered using two large steering knobs (Fig. 1) that steer the tip using Bowden cables. Only the tip of the colonoscope (+/$-$8 cm) can thus be controlled actively, the flexible shaft of the endoscope follows passively [7]. This 60-year-old non-ergonomic control section also causes physical complaints [8], a consequence that reduces the colonoscopic capacity while demand rises. With our research, we aim to improve intuitiveness and ergonomics of the endoscope. We are focusing on colonoscopy because of the clear demand.

A typical colonoscopy procedure consists of a retrograde insertion phase and a retraction phase. The insertion is done as quickly as possible until the beginning of the organ, the cecum, is reached. Retraction has a recommended duration of at least 6 min [9]. During this phase, the colon wall is inspected for anomalies which are removed if necessary [7, 10]. Commonly found anomalies are so-called polyps, uncontrolled growth of the mucosa on the colon wall. Due to a minimal retraction time, procedure efficiency and colonoscopy capacity can only be improved by shortening the insertion phase. An easier control mechanism is expected to make endoscope insertion faster and the learning curve shorter.

Robotic systems with intuitive controllers such as a remote joystick have been shown to reduce the experienced workload and improve control intuitiveness for endoscopists [11]. Image-based navigation may help to improve intuitiveness of robotic systems even further [12]. During the insertion phase, image-based navigation could be useful in finding the target direction and steering toward it automatically. The colon is visible with a colonoscope as a tubular, folded structure. The target of the colonoscope almost always is the deepest visible area, often corresponding to the center of the lumen. This area usually presents as the darkest area in the endoscopic images, which is a useful feature for image-based navigation.

Central lumen detection for automatic endoscope steering has been investigated before [13–17]. Automated endoscope steering was reviewed as well [12]. Most of the research in this area focuses on segmenting the central lumen area as accurately as possible. Although accurate central lumen detection in colonoscopic images is technologically feasible, none of the mentioned systems to our knowledge are clinically accepted or even tested for clinical applicability.

All previous techniques are based on the assumption that by centralizing the lumen, the colonoscope will travel the right path through the colon. Complicating factors herein are image artifacts, such as fluids or bubbles on the lens, which make images hard to interpret. The lens may also be pressed against the colon wall, causing a ‘red-out’ or ‘wall view’. Additionally, the camera can be moved substantially between frames, causing motion blur artifacts [13]. These complicating factors and artifacts have impeded successful implementation of this technique up to now. Moreover, centralizing the lumen is not always desired by the endoscopist. Sometimes, maneuvers using the colonic wall are performed on purpose to advance the endoscope further [18, 19].

We have developed and evaluated a new algorithm for colonoscopy steering based on dark region centralization [20]. This algorithm is implemented in an *assisting* fashion and *predicts* whether images will contain useful information. The prediction diminishes the influence of artifacts. This algorithm was adapted to be implemented in a robotized flexible endoscopy system called Teleflex [21, 22]. The vision-based functionality is meant to assist during procedures and can be actively turned off and on by the endoscopist.

The aim of the current study was to evaluate the assisting automated lumen centralization algorithm in terms of technical feasibility in a clinical setting. Clinical feasibility means that the system enables colonoscopy that is at least as efficient and effective as the conventional method (non-inferiority), but this was reported on elsewhere [23]. Technical feasibility is defined as the system’s performance during colonoscopy and includes user feedback on system functionality. The emphasis of this study is on the technical performance of the robotic system as a whole.

## Materials

**Fig. 2:**
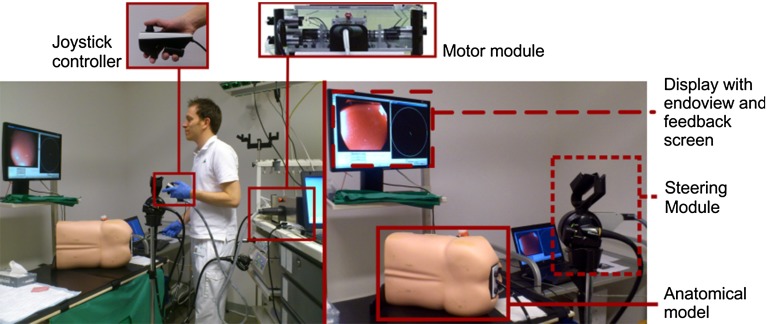
The robotized experimental setup for this study. The endoscopist looks at the display and controls the endoscope with the joystick to perform a colonoscopy on the anatomical model. The robotic parts were removed during conventional steering

Experts in endoscopy and inexperienced participants performed colonoscopy on a simulated colon model using either the conventional endoscopic steering method (turning the steering knobs) or the robotized setup (Fig. 2).

### Robotic setup with target centralization algorithm

There were three main requirements for control of the complete system. First, real-time functionality was essential. The procedure needed to be executable without the endoscopist having to wait for visual feedback from the system. Second, the endoscopist needed to be able to overrule the algorithm instantaneously at any point. Third, the complete functionality of the system needed to be intuitive, which means it should be easily learned and implemented in clinical practice. In our system, control of the tip in the robotized setup could be established either through remote user input (e.g., a joystick device) or through the image-based navigation algorithm.

**Fig. 3:**
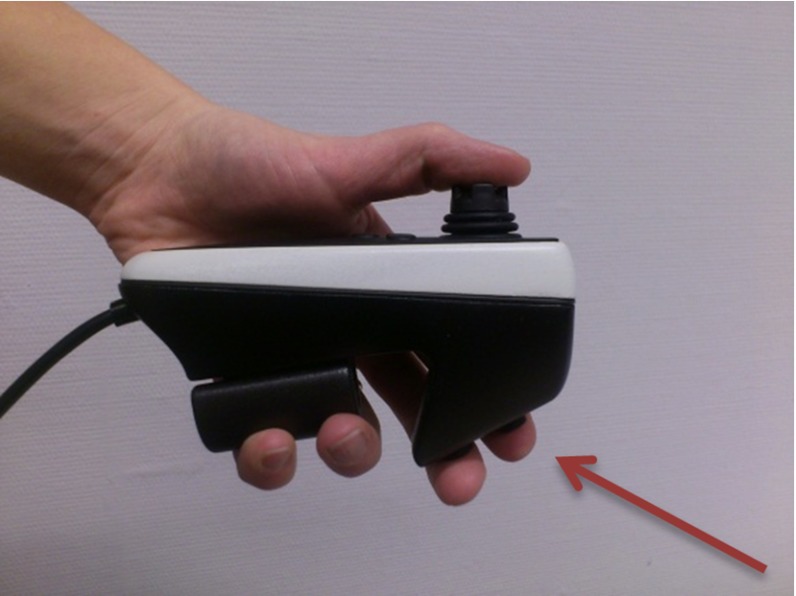
The button to enable automated navigation could be pressed by the forefinger

**Fig. 4:**
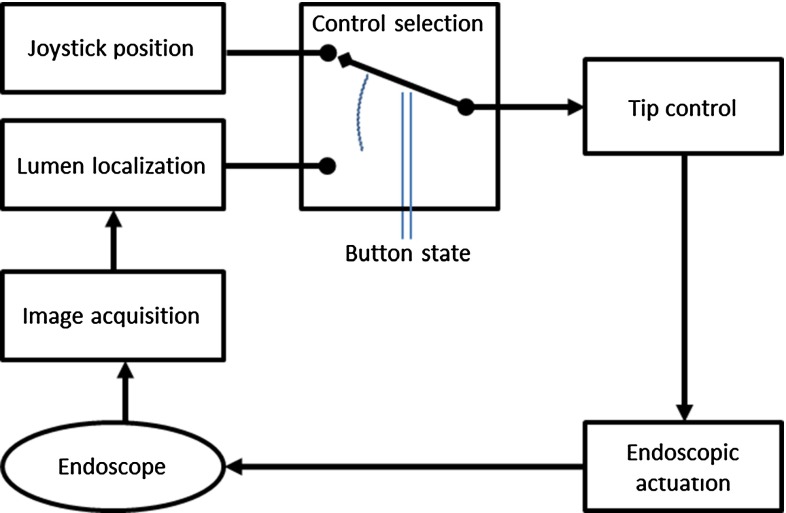
Control loop of the robotic flexible endoscope system that was used in this study

An algorithm was developed with these requirements in mind and implemented in a robotized endoscopy system [21]. In the robotized endoscope setup, the tip of the endoscope is controlled through telemanipulation using a joystick controller (Fig. 3). This interface has been validated before [11, 24–26]. If a designated button was pressed and held on the joystick controller (arrow in Fig. 3), steer commands were generated by the navigation algorithm. The user thus had to actively choose if the steering would be controlled by the algorithm. This assisting functionality ensured an immediate overrule option and therefore future patient safety. For both types of input commands, the same control loop was passed in real-time (Fig. 4) [27].

**Fig. 5:**
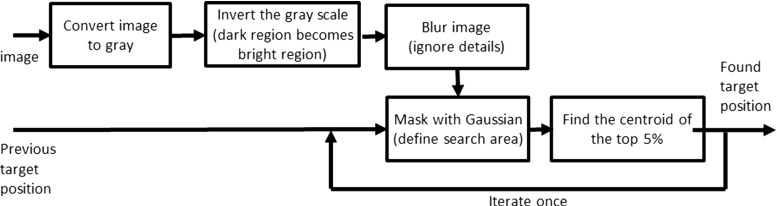
Flowchart of algorithm steps. The current image and the previous target are used as input to find the current target location

**Fig. 6:**
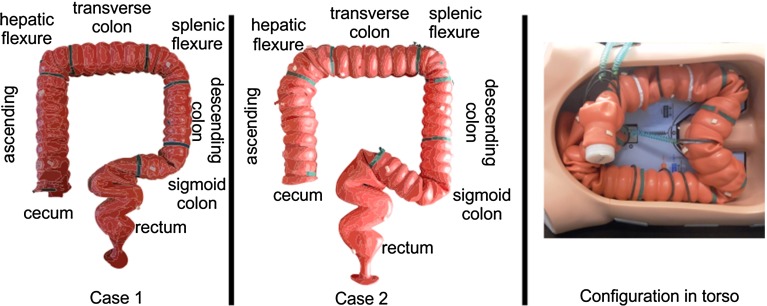
Case configurations 1 (*left*, novices) and 2 (*middle*, experts). Anatomical names for colon segments are also illustrated per case. Segments are bordered by the *green lines* to ensure logging consistency between experiments. On the *right*, the configuration inside the torso during the experiments is shown. This configuration was invisible to participants

The robotic setup consisted of a conventional flexible endoscope with the handle linked to the steering module [21, 24]. The handle and the steering module were suspended on a custom-designed holder. The tip of the endoscope could then be manipulated through motor control, enabled by the joystick controller and a standard laptop, while shaft insertion and rotation were done manually.

The assisting navigation algorithm’s main task was to detect the target of the endoscope, which was the lumen center, through image analysis. It used information from previous images to estimate the target location and corrected with the current image information (Fig. 5). Let $f_i(\mathbf y )$ be an image sequence with frame index *i* and pixel positions $\mathbf y$. The estimate of the pixel position of the lumen center $\mathbf {x}(i)$ of frame *i* will be called $\hat{\mathbf{x }}(i)$. $\hat{\mathbf{x }}(i)$ is obtained following the CoG computation in [27]. All other frames are processed as displayed in the flowchart (Fig. 5). To suppress noise influence, Gaussian low-pass filtering is applied, and subsequently the image is inverted. Then, a maximum needs to be found instead of a minimum. The Gaussian convolution ensures windowing the maximum toward the previously estimated target position. Iteration was applied to increase the bias the Gaussian convolution causes, meaning larger shifts of the target between frames still resulted in accurate target estimation. Performance of the algorithm was evaluated in a previous study using human colonoscopy images [20].

Participants were asked to perform a procedure on a plastic, earlier validated, anatomical model (Kyoto Kagaku, Kyoto, Japan) [28]. For each configuration, 21 foam ‘polyps’ were applied on the inside of the colon. The polyps corresponded in size and location to the polyps described in [29].

### Methods

Eight expert endoscopists (each performed ${>}1000$ endoscopic procedures) and ten inexperienced technical medicine students [without experience in endoscopy but with knowledge of anatomy, physiology and pathology of the colon and abdomen (novices)] performed a simulated colonoscopy on the plastic model of the colon. The participants were asked to perform a colonoscopy twice: once using the conventional steering knobs and once using the robotic setup with the assisting target centralization function (automated target centralization, ATC). The order in which they performed the procedures was randomized: Half of the participants started with the robotic method and half of the participants with the conventional method. They were asked to intubate the endoscope as fast as possible and retract in 6 min while inspecting the bowel wall for lesions. Afterward, participants were asked for their subjective opinion by means of a questionnaire.

**Fig. 7:**
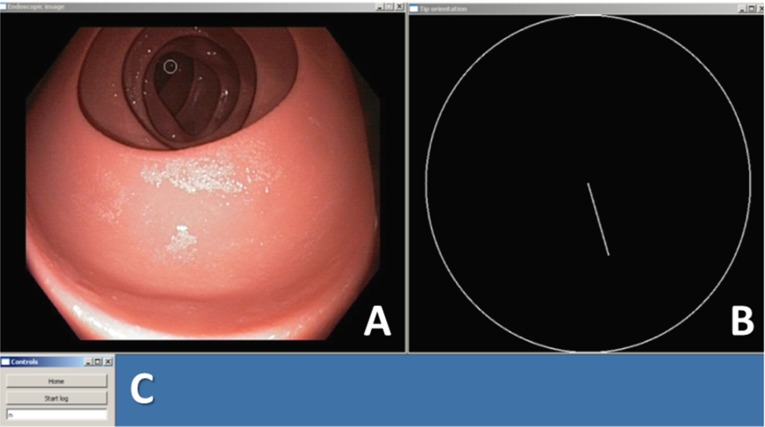
Screenshot of a representative situation during the procedure. In the endoview (A), a small white circle indicates the target position in the image. The tip bending diagram shows the current amount and direction of tip bending (B). A small window, needed for logging and changing settings during the procedure, was continuously present but could be ignored by the test participants (C)

Novices performed a colonoscopy with the simplest colon configuration (case 1, Fig. 6). Experts performed the experiments on a more complicated configuration because of their experience with the colonoscopy procedure itself (case 2, validated in [28]). This distinction was made after pretesting both groups. Novices were not able to complete case 2 with any of the two modalities, while experts were unrealistically fast in completing case 1. This did not hinder study evaluation because the performance was not compared between groups, but between modalities. The polyps inside the model were to be detected upon retraction of the endoscope to determine the competence in anomaly detection.

Participants continuously received visual feedback of the endoscopic image and feedback of the tip bending state when using the robotic setup, as depicted in Fig. 7. In the left pane (A), a small white circle depicts the target found by the algorithm in the endoscopic image. When using the robotic setup, the participant could choose to press a button and activate ATC if the position of this circle corresponded to the desired steering direction. This circle was always visible, even during the conventional colonoscopic procedures. The right pane (B) shows the amount and direction of tip bending, currently illustrating a tip that is bent halfway downward.

All participants used the same Olympus CF180 colonoscope connected to an Olympus CV-180 Evis Exera II video processor. This type of colonoscope produces 576,768 pixel images with 25 frames per second. It has a field of view of $170^{\circ }$ and a field depth of 3–100 mm. The ATC algorithm was implemented in Python 2.7 [30] using OpenCV [31] on a standard Windows laptop (Dell Probook 6560b). Analyses were done using IBM SPSS Statistics 20 (IBM, Armonk, NY, USA) and MATLAB R2011b (The Mathworks Inc., Natick, MA, USA).

### Evaluation parameters and statistical analysis

Main evaluation parameters were as follows: the percentage of time using ATC (ATC use, % of total), the time ATC was on (TO, number of times), the ‘on’-time per period (DUR, in frames) and subjective user feedback for technical feasibility.

Technical feasibility focuses on the use of the system and can be compared between the two participant groups. The colon was divided into seven segments (Fig. 6), and transition of the endoscope from one segment to the other was timed. This was done to enable parameter evaluation per segment. The earlier clinical evaluation was done to compare the different colonoscopy methods, and therefore, the comparison was made within the two participant groups.

It was expected that the ATC would predominantly be used in the longer, straight segments of the colon. In these areas, the view on the lumen is optimal which would mean the algorithm and the user should agree. We also hypothesized that if ATC would be turned ‘on’, the introduction would be faster than without centralization.

Overall statistical evaluation was done, if relevant, using a Mann–Whitney *U*-test with a significance level *p* of 0.05. In some cases, the Mann-Whitney *U*-test could not be used since there was no variance homogeneity. Those cases are clearly indicated in the “Results” section, together with the test that was used and the significance level.

## Results

The technical feasibility results of the system are described below. For completeness, a summary of the clinical results is provided as well.

### Technical feasibility: use of the system

**Fig. 8:**
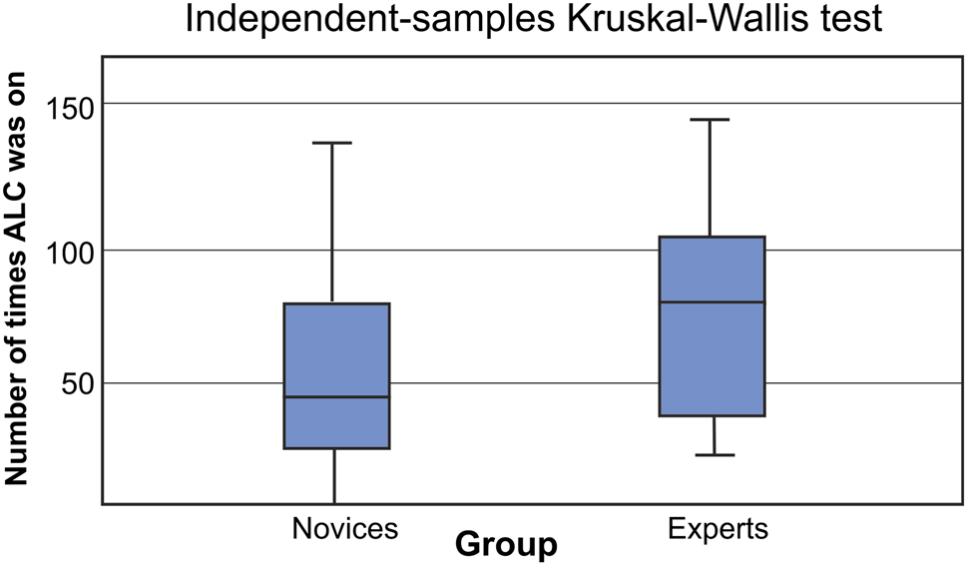
Number of times that automatic lumen centralization was turned on (*y*-axis) per group (*x*-axis). Note that a Kruskal–Wallis test was used (significance level *p* of 0.05)

ATC was turned on by the novices for a median of 4.2 % of the time during insertion and 0.3 % during retraction. Experts turned ATC on for 4.0 % of the time during insertion and 11.6 % during retraction. The number of times ATC was turned on was 77 times per procedure for the experts and 59 for the novices (Fig. 8).

**Fig. 9:**
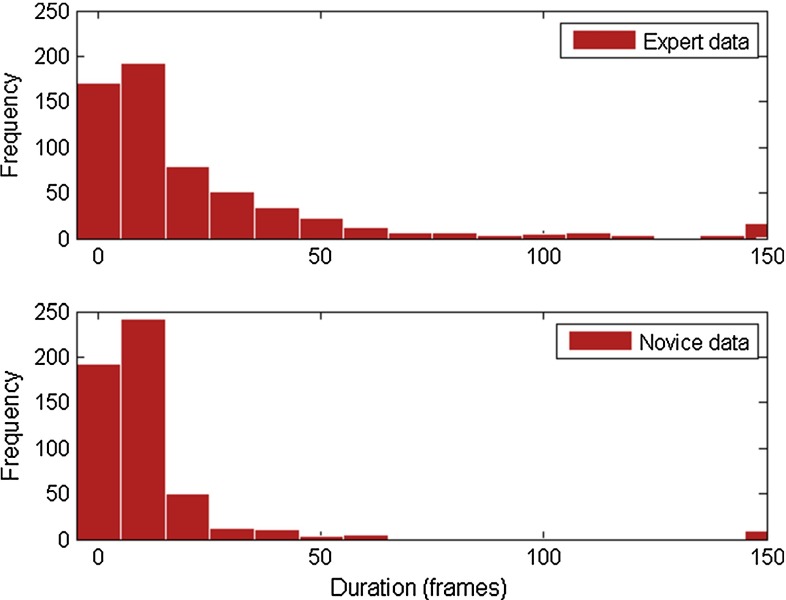
Histogram showing mean duration of ATC use (DUR) for experts (*top*) and novices (*bottom*)

The mean duration of ATC use (DUR) was significantly longer in the expert group than in the novices group $(p < 0.001)$. Median DUR was 12 frames for experts and 7 frames for novices (Fig. 9).

#### Subjective questionnaire results

All novices and three experts thought that endoscope insertion was easier with the robotic system with ATC. However, at least four experts indicated that they expect additional value of the ATC functionality during retraction. The easy rotation of the endoscope tip with the joystick, used for colon wall lesion inspection, became even easier when the tip could be centralized automatically, so when ATC was enabled. Almost all users agreed that the robotic system would make performing a colonoscopy easier for novices (10 novices, 7 experts); 50 % of the experts and all novices were positive about the platform. An additional finding (not in the questionnaire) was that during the experiments, some test persons indicated that they ‘followed the white circle’ while inserting the endoscope during the conventional procedures.

### Clinical feasibility: summary

All included participants reached the cecum using both steering methods. Novices showed no significant differences in time to cecum (TTC) between using conventional (median 11 min 47 s, with Q1–Q3 8 min 19 s – 15 min 33 s) or robotic control (median 8 min 56 s, Q1–Q3 6 min 46 s – 16 min 34 s, $p = 0.65$). Experts showed a trend toward a faster introduction using the conventional method (median 2 min 9 s, Q1–Q3 1 min 13 s – 7 min 28 s) than with the robotic method (median 13 min 1 s, Q1–Q3 5 min 9 s – 16 min 54 s, $p = 0.12$).

The significant results of the time analyses per segment are listed in Table 1. Novices were significantly faster during insertion through the descending colon with the robotic setup and assisting algorithm. Experts were significantly faster in many segments using the conventional method, but not in the descending colon and the splenic flexure.

**Table 1:** Time comparison per colon segment (only significantly differing segments listed, lowest value formatted bold)

Segment (insertion)	Median robotic (s)	Median conventional (s)	Significance level (*p*)
*Novices*			
Descending colon	**16**.**12**	19.66	0.04
*Experts*			
Rectum	87.14	**33**.**40**	0.02
Sigmoid colon	103.68	**34**.**48**	0.02
Transverse colon	225.48	**21**.**72**	0.03
Hepatic flexure	16.68	**3**.**08**	0.01
Ascending colon	14.80	**4**.**48**	0.03

Novices found slightly more polyps (88.1 %, Q1–Q3 79.8–95.2 %) with the robotic method compared to using the conventional system (78.6 %, Q1–Q3 75.0–91.7 %, nonsignificant). Experts found significantly more polyps using the conventional method with a median detection rate of 81.0 % (Q1–Q3 76.2–85.7 %) against 69.0 % (Q1–Q3 61.0–75.0 %, $p = 0.02$) when using the robotic method with ATC. The retraction times of the experts were within the range of 3.42–6.15 min. One outlier was present, which was an expert using the conventional setup (${>}8.5$ min). For novices, this range was 3.83–7.64 min.

**Fig. 10:**
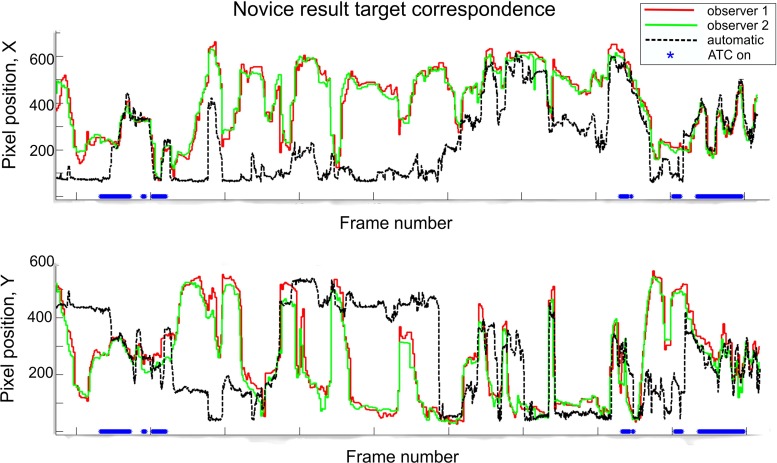
Example graph showing *X* (*top*) and *Y* (*bottom*) pixel coordinates of the found target in the image. The *dashed line* indicates the automatically found target, and the others show the manually indicated target. The *straight lines at the bottom of each graph* indicate periods that ATC was turned on

## Discussion

In this study, we evaluated the technical feasibility of an assisting automated lumen centralization algorithm implemented in a robotized colonoscopy setup. Our hypothesis was that the ATC would work in real time in the robotized system it was implemented on, that it would predominantly be used in straight segments of the colon and that it would make endoscope introduction faster.

Clinically, we showed that novices were at least as efficient with the robotic system as with the conventional one, with a trend toward faster introduction. A significant difference was shown in a straight segment of the colon (descending colon). However, because of the low ATC use percentage, it is uncertain whether this faster time is solely due to ATC use. Expert colonoscopists are fully trained using a conventional endoscope, and therefore, it was expected that they are faster (TTC) using the conventional method. However, in both straight and curved segments, the robotic setup also obtained equal results compared to the conventional setup in terms of clinical efficiency. From this, we expect performance with the new system to improve with more training.

The system was developed to support endoscope insertion and was expected to be easier and faster in this part of the procedure. Interestingly however, experts used ATC almost 12 % of the time during the *retraction* phase of the procedure while this was not the purpose of the developed application. When asked for their opinion, all expert users and some of the novices indicated that they experienced *real additional value of the ATC functionality* during retraction. It was considered more intuitive to centralize the lumen with the robotic system during the wall inspection for possible lesions than with the conventional system. Furthermore, all users indicated to see added value of the system *for novice users*, which is considered an excellent result.

Study results also revealed interesting comments on the visual cue that indicated the automatically found target direction. Several novice and expert users not only deemed the little white circle helpful, but stated they were following the cue even when they were not using the ATC function. This implies that the added functionality of the ATC algorithm may be partly established by visual assistance and partly by autonomous correction of the tip.

Experts not only used automatic lumen centralization more often (although nonsignificant), but also for a longer period of time ($p < 0.001$, Figs. 8, 9). Yet, the numbers overall are lower than expected. The low percentage of time ATC was used could theoretically be due to the low level of agreement of the users with the target location of the central lumen. Therefore, we compared the automatically found target location to the location that medical experts would steer the endoscope. Figure 10 illustrates the *X*- and *Y* coordinates of the found targets during part of a novices’ introduction phase. The solid lines indicate manual indication of the target by expert observers, and the dashed line shows the automatic result. If ATC is turned on the lines correspond well. However, there also are regions where the lines correspond well and ATC is not turned on. Therefore, we think that lack of training with the system causes the low use of ATC, which should therefore be improved in a next study. The fact that the ATC is not always ‘right’ can be due to a number of factors, including the fact that the medical doctor would not always steer toward the central lumen. This is the reason the ATC functionality is designed in an assisting fashion.

Measuring clinical effectiveness was done by adding the polyp detection. Novices found an equal amount of polyps on average in both modalities; experts found significantly more polyps using the conventional system. However, this measure was suffering from several confounding factors, such as differing retraction times. We think that clinical effectiveness therefore cannot be established reliably from this outcome measure.

In terms of technical feasibility, the algorithm functions well and has helpful assisting functionality. A next study should be optimized specifically on the amount of training users need to perform equally with the new system and the conventional one.

## Conclusion

In conclusion, the ATC algorithm combined with the Teleflex robotized endoscope setup works well in an experimental setup that closely resembles the clinical environment. The non-inferiority of this system is shown for novices, although ATC use was lower than expected. The relatively extensive use of ATC during the retraction phase of the procedure suggests a possibly interesting added value of the system during this phase, although this needs to be investigated further.

The next steps in this research include adjusting the system according to user feedback and an elaborate study on clinical effectiveness using thoroughly trained participants.

The developed algorithm was tested in a colonoscopy simulation environment, but was not specifically designed for this. The algorithm can therefore be applied in any robotic flexible endoscopy system that is used in diagnosis of tubular organs.

## Electronic supplementary material

Supplementary material 1 (avi 3580 KB)

## References

[1] Siegel R, Desantis C, Jemal A (2014). Colorectal cancer statistics, 2014. CA Cancer J Clin.

[2] Autier P (2013) Colorectal cancer screening works ; ‘irrefutable’ evidence that fall in death rates is attributable to screening programmes screening for colorectal cancer (CRC) in European countries is highly effective in reducing mortality, ECC 2013 press release. http://www.esmo.org/Conferences/Past-Conferences/European-Cancer-Congress-2013/News/ECC-2013-Press-Release-Colorectal-Cancer-Screening-Works-Irrefutable-Evidence-that-Fall-in-Death-Rates-is-Attributable-to-Screening-Programmes. Accessed 01 Nov 2014

[3] Pedrosa MC, Farraye FA, Shergill AK, Banerjee S, Desilets D, Diehl DL, Kaul V, Kwon RS, Mamula P, Rodriguez SA, Varadarajulu S, Song L-MWK, Tierney WM (2010). Minimizing occupational hazards in endoscopy: personal protective equipment, radiation safety, and ergonomics. Gastrointest Endosc.

[4] Geers G, van der Vlis-Vester E (2012) Screen: special darmkanker. DRD Support BV, Amsterdam

[5] van Veldhuizen-Eshuis H, Carpay MEM, van Delden JA, Grievink L, Hoebee B, Lock AJJ, Reij R (2011) Uitvoeringstoets bevolkingsonderzoek naar darmkanker. Rijksinstituut voor Volksgezondheid en Milieu (RIVM), Den Haag

[6] Vargo JJ (2010). North of 100 and south of 500: where does the ‘sweet spot’ of colonoscopic competence lie?. Gastrointest Endosc.

[7] Waye JD, Rex DK, Williams CB (2009). Colonoscopy: principles and practice.

[8] Shergill AK, McQuaid KR, Rempel D (2009). Ergonomics and GI endoscopy. Gastrointest Endosc.

[9] Barclay RL, Vicari JJ, Doughty AS, Johanson JF, Greenlaw RL (2006). Colonoscopic withdrawal times and adenoma detection during screening colonoscopy. N Engl J Med.

[10] Obstein KL, Valdastri P (2013). Advanced endoscopic technologies for colorectal cancer screening. World J Gastroenterol.

[11] Rozeboom E, Ruiter J, Franken M, Broeders I (2014). Intuitive user interfaces increase efficiency in endoscope tip control. Surg Endosc.

[12] van der Stap N, van der Heijden F, Broeders IAMJ (2013). Towards automated visual flexible endoscope navigation. Surg Endosc.

[13] Krishnan SM, Tan CS, Chan KL (1994) Closed-boundary extraction of large intestinal lumen. In: Proceedings of the 16th annual international conference of the IEEE. Engineering in medicine and biology society, pp 610–611

[14] Xia S, Krishnan SM, Tjoa MP, Goh PMY (2003). A novel methodology for extracting colon’s lumen from colonoscopic images. J Syst Cybern Inform.

[15] Zhiyun X (2000). Computerized detection of abnormalities in endoscopic oesophageal images.

[16] Asari KV (2000). A fast and accurate segmentation technique for the extraction of gastrointestinal lumen from endoscopic images. Med Eng Phys.

[17] Gillies D, Khan G (1996). Vision based navigation system for an endoscope. Image Vis Comput.

[18] Hansel SL, Prechel Ja, Horn B, Crowell MD, DiBaise JK (2009). Observational study of the frequency of use and perceived usefulness of ancillary manoeuvres to facilitate colonoscopy completion. Dig Liver Dis.

[19] Shah SG, Saunders BP, Brooker JC, Williams CB (2000). Magnetic imaging of colonoscopy: an audit of looping, accuracy and ancillary maneuvers. Gastrointest Endosc.

[20] van der Stap N, Slump CH, Broeders IAMJ, van der Heijden F (2014) Image-based navigation for a robotized flexible endoscope. Lecture Notes in Computer Science, vol 8899. Springer, Switzerland, pp 77–87

[21] Ruiter J, Rozeboom E, Van Der Voort M, Bonnema M, Broeders I (2012) Design and evaluation of robotic steering of a flexible endoscope. In: IEEE BioRob, pp 761–767

[22] Ruiter JG, Van Der Voort MC, Bonnema GM (2013) User-centred system design approach applied on a robotic flexible endoscope. In: Conference on systems engineering research (CSER’13)

[23] Pullens HJM, Van Der Stap N, Rozeboom ED, Schwartz MP, Van Der Heijden F, Van Oijen MGH, Siersema PD, Broeders IAMJ (2015) Colonoscopy with robotic steering and automated lumen centralization: a feasibility study in a colon model. Endoscopy, Georg Thieme Verlag KG Stuttgart, New York. doi:10.1055/s-0034-1392550 10.1055/s-0034-1392550 26126158

[24] Rozeboom ED, Broeders IAM, Fockens P (2015). Feasibility of joystick guided colonoscopy; assessing the learning curves of experts and novices. J Robot Surg.

[25] Rozeboom ED, Ruiter JG, Franken M, Schwartz MP, Stramigioli S, Broeders IAMJ (2014). Single-handed controller reduces the workload of flexible endoscopy. J Robot Surg.

[26] Kuperij N, Reilink R, Schwartz MP, Stramigioli S, Misra S, Broeders IAMJ (2011) Design of a user interface for intuitive colonoscope control. In: IEEE international conference on intelligent robots and systems 2011,pp 2076–2082

[27] Reilink R, Stramigioli S, Misra S (2010) Image-based flexible endoscope steering. In: IEEE/RSJ international conference on intelligent robots and systems, no i, p 6

[28] Plooy AM, Hill A, Horswill MS, Cresp ASG, Watson MO, Ooi S-Y, Riek S, Wallis GM, Burgess-Limerick R, Hewett DG (2012). Construct validation of a physical model colonoscopy simulator. Gastrointest Endosc.

[29] Gralnek IM, Carr-Locke DL, Segol O, Halpern Z, Siersema PD, Sloyer A, Fenster J, Lewis BS, Santo E, Suissa A, Segev M (2013). Comparison of standard forward-viewing mode versus ultrawide-viewing mode of a novel colonoscopy platform: a prospective, multicenter study in the detection of simulated polyps in an in vitro colon model (with video). Gastrointest Endosc.

[30] Python Software Foundation (2014) Python v2.7 documentation. Documentation. https://docs.python.org/2.7/

[31] OpenCV development Team (2014) OpenCV documentation. Documentation. http://docs.opencv.org/

